# Anti-Obesity Effect of *Erigeron annuus* (L.) Pers. Extract Containing Phenolic Acids

**DOI:** 10.3390/foods10061266

**Published:** 2021-06-02

**Authors:** Yulong Zheng, Yoon-Hee Choi, Ji-Hyun Lee, So-Yeon Lee, Il-Jun Kang

**Affiliations:** 1 Department of Food Science and Nutrition & the Korean Institute of Nutrition, Hallym University, Chuncheon 24252, Korea; zyl1994@naver.com (Y.Z.); leejihyun@hallym.ac.kr (J.-H.L.); syeon300@hallym.ac.kr (S.-Y.L.); 2 Department of Food and Nutrition, Hallym Polytechnic University, Chuncheon 24210, Korea; cyh@hsc.ac.kr

**Keywords:** C57BL/6J mice, high-fat diet, anti-obesity, AMPK pathway, phenolic acid, *Erigeron annuus* (L.) Pers

## Abstract

*Erigeron annuus* (L.) Pers. water extract (EAW) was investigated for its anti-obesity effects in C57BL/6J mice on a high-fat diet. Mice were divided into groups fed normal and high-fat diets (ND and HFD, respectively), and HFD mice were treated with EAW (50, 100, and 200 mg/kg/day) for 8 weeks. Inhibition of HFD-induced obesity by EAW was evaluated using biochemical parameters, immunohistochemistry, real-time PCR, and immunoblot assay. EAW supplementation significantly diminished the final body weight, adipose tissue size, and epididymal adipose tissue volume compared with mice with obesity induced by HFD (*p* < 0.05 for all). EAW also decreased serum triglyceride (TG) and LDL-cholesterol (LDL-c) levels in obese mice. EAW attenuated HFD-induced obesity by down-regulating C/EBPα, PPARγ, and SREBP-1c to suppress adipogenesis. Moreover, this study indicated that EAW activates the AMPK pathway and increases ACC phosphorylation and downstream CPT1 expression in HFD-induced obese mice. Furthermore, several phenolic acids with anti-obesity properties have been identified in EAW, including quinic acid, caffeic acid, chlorogenic acid, and 3,4-dicaffeoylquinic acid. Based on these data, EAW has anti-obesity effects in vivo, which indicates that it is an excellent candidate for the development of anti-obesity functional foods.

## 1. Introduction

The number of overweight or obese people is rapidly increasing due to long-term sedentary habits, low prices for high-calorie diets, a lack of physical activity, and the rapid Westernization of lifestyle [1]. Obesity is described as a state in which excess fat accumulates in the body caused by unbalanced energy intake and consumption. Obesity can lead to the accumulation of epicardial fat, pancreatic fat, liver fat with fat poisoning, and insulin resistance [2]. Since obesity causes changes in the internal milieu of the human body, the incidence of hypertension, hyperlipidemia, cardiovascular atherosclerosis, and metabolic syndrome is increasing [3]. Obesity is becoming a severe health problem worldwide as has been recognized as the cause of much morbidity. Therefore, researchers are making various efforts to treat or prevent obesity. Sibutramine (Reductil), Orlistat (Xenical), and thermogenic agents are currently the most active obesity treatments. Although the FDA has approved these drugs for treating obesity, they have been reported to cause side effects such as stool or urinary incontinence and impaired absorption of fat-soluble vitamins [4]. Therefore, we have focused more attention on natural remedies for obesity, which have fewer side effects and may be relatively inexpensive [5].

The vital metabolic sensor adenosine monophosphate (AMP)-activated protein kinase (AMPK) can regulate energy balance at the cellular and whole-body level [6]. AMPK stimulates adenosine triphosphate (ATP) production pathways (such as fatty acid oxidation) and blocks the ATP consumption assimilation pathway (such as energy-consuming fat production) [7]. In addition, triggering AMPK prevents adipogenesis and adipocyte differentiation-related gene expression, such as PPARγ, fatty acid synthase (FAS), and adipocyte protein 2 (aP2) [8]. Therefore, AMPK activators have attracted much interest as therapeutics to improve diabetes and obesity-related metabolic diseases [9].

*Erigeron annuus* (L.) Pers. (EA) has been reported to contain a variety of phenolic acids and their derivatives [10,11] and is used in traditional medicine to treat acute infectious hepatitis, acute gastroenteritis, hematuria, and indigestion [10,12]. Natural plant foods usually contain a lot of bioactive compounds as antioxidants [13]. Different components of EA were recently observed to possess potent DPPH radical scavenging and antioxidative activity [14]. EA leaf extracts containing caffeic acid showed antioxidant activity by ABTS and FRAP assays [15]. EA flower extracts exhibit anti-inflammatory effects and inhibit the secretion of nitric oxide synthase in inflammation induced by lipopolysaccharides in macrophages [16]. Furthermore, EA protected neuronal cell cytotoxicity induced by H_2_O_2_ in MTT and lactate dehydrogenase tests [15]. Although EA has many compounds and biological activities, analyzing and comparing the potential of EA as a cheap food to improve and control obesity in vivo has not been reported. For that reason, we have examined the effects of *Erigeron annuus* (L.) Pers. water extract (EAW) on HFD-induced obesity in mice and discussed the anti-obesity mechanism of EAW from aspects related to the AMPK pathway, mRNA levels, and genetic factors related to lipid metabolism.

## 2. Materials and Methods

### 2.1. Material

EA samples were collected locally in Chuncheon, Gangwon-do, Korea, and naturally dried at a room temperature of 15 °C. The dried leaves and stems were crushed and extracted with hot water for 3 h, and this was repeated three times. The extract was concentrated with a vacuum evaporator (Rotavapor R-220, Buchi, Flawil, Switzerland), followed by freeze-drying with a freeze dryer (Il Shin Lab Co., Seoul, Korea) to obtain 21.8 g of powder. The extraction yield of EAW was 27.3%.

### 2.2. Experimental Animals

5-week-old male C57BL/6J mice (Central Lab. Animal, Inc., Seoul, Korea) were used as experimental animals. All procedures were approved by the Animal Experimental Ethics Committee of Hallym University (Permit Number: Hallym 2018-61). Animals were adapted for 2 weeks to a 12h light/dark cycle, with a relative humidity between 45%~55% and temperature between 20.9~22.6 °C. The mice were divided into 6 groups, with 8 mice in each group. One group received a 10% fat diet as a normal diet (ND), and the remaining groups received a 60% fat diet as HFD groups. Three HFD groups received oral EAW at concentrations of 50, 100, and 200 mg/kg/day. Another HFD group was orally administered 100 mg/kg/day of *Garcinia cambogia* extract (G100) daily as a positive control due to the weight loss effects of *Garcinia cambogia* extract [17]. Meanwhile, the remaining ND and HFD groups were taken orally with distilled water used to dissolve the extract. During the 8-week experimental period, body weight was measured once a week and dietary intake twice a week. Before orbital blood sampling from the animals anesthetized with 2,2,2-tribromoethanol and 2-methyl-2-butanol (Sigma-Aldrich, MO, USA), the experimental animals were fasted for 12 h and euthanized by cervical dislocation after blood collection. Biochemical tests were performed on the collected blood samples to analyze serum biomarkers. The animals were sacrificed and dissected to obtain liver and epididymal adipose tissue, washed with physiological saline, and stored immediately at −70 °C.

### 2.3. Measurement of Serum Biochemical Parameters

Blood samples were collected and centrifuged at 3500× *g* for 20 min at room temperature. The obtained serum was stored at −70 °C. Serum levels of triglycerides (TG), LDL-cholesterol (LDL-c), HDL-cholesterol (HDL-c), glucose (GLU) alanine aminotransferase (ALT), and aspartate aminotransferase (AST) were measured by KoneLab 20 (Thermo Fisher Scientific, Waltham, MA, USA).

### 2.4. RNA Extraction and Real-Time PCR

Total RNA of epididymal adipocytes and hepatocytes were extracted according to the protocol of Easy-Blue (iNtRON Biotechnology, Seongnam), and total RNA was quantified with NanoDrop (NanoDrop 2000c, Thermo Scientific, Waltham, MA, USA). Then, 1 µg of RNA was transformed into cDNA using the Roche Applied Science cDNA Synthesis Kit (Penzberg, Germany). Table 1 shows the primers used. Real-time quantitation was accomplished using a LightCycler 480 (Roche Diagnostics, Mannheim, Germany), and the PCR reaction mixture was accomplished by the LightCycler 480 SYBR Green I Master system (Roche, Germany). Rear-time PCR conditions were as follows: 95 °C for 10 min followed by forty-five cycles at 95 °C for 15 s, 60 °C for 5 s, and 72 °C for 15 s. As a result of continuous 3 or 4 replicates, GAPDH was used as an internal regulator to standardize the volume change of the template.

### 2.5. Analysis of Protein Level

Liver and epididymal adipose tissues were homogenized with PRO-PREP lysis buffer (iNtRON Biotechnology, Seongnam, Korea), and BCA protein assay was used to quantify the lysate (Scientific, Loughborough, UK). Then, 20 μg of the protein extract was heated with 2X sample buffer at 95 °C for 5 min and loaded on 10%-SDS-PAGE gels. The sample was electrophoresed at 100 V for 90 min and then transferred to a PVDF membrane. The membrane was blocked by 1× TBST supplemented with 5% skim milk and incubated overnight with the primary antibody at 4 °C; after that, it was incubated with the secondary antibody at room temperature for 1 h. A rinse was performed 3 times with TBST for 10 min before each stage. Antibodies to PPARγ, C/EBPα, SREBP-1c, adiponectin, phosphorylated-AMPKα (p-AMPK), AMPK, acetyl-CoA carboxylase (ACC), p-ACC, and carnitine palmitoyltransferase I (CPT1) were obtained from Cell Signaling Technology (Beverly, MA, USA). The imaging software was subjected to substrate analysis according to the manufacturer’s Forte Western HRP protocol (Millipore Japan, Tokyo, Japan) to analyze the density of specific bands.

### 2.6. Adipose Histological Analysis 

Epididymal adipose tissue was fixed with 4% paraformaldehyde, and the paraffin-embedded block was sectioned using a microtome with a 5 µm thickness. The paraffin sections were stained with hematoxylin and eosin (H and E staining) according to standard procedures [18]. The area of adipocytes was estimated by Adiposoft software (National Institutes of Health, Bethesda, MD, USA).

### 2.7. LC-ESI-MS and LC-ESI-MS/MS Analysis

The extracts were separated using a Shiseido HTS HPLC system (Shiseido, Tokyo, Japan). Chromatographic separation was performed on a Phenomenex Kinetex C18 column (2.1 × 100 mm, 2.6 μm), thermostatted at 40 °C and then pre-equilibrated in solvent A (0.1% formic acid in water), and extracts were eluted with increasing percentages of solvent B (acetonitrile with 0.1% formic acid) at a flow rate of 0.5 mL/min. The elution gradient steps were as follows: 0–1 min, 0% B; 1–4 min, gradient to 70% B; 4–6 min, 100% B; 6–6.2 min, gradient to 0% B; and 6.2–10 min, 0% B. Mass detection was performed in an API3200 QTRAP equipped with an electrospray ionization (ESI) source and a triple quadrupole-ion trap spectrometer controlled by Analyst 5.1 software. The negative ionization mode was applied, and the optimized instrument settings were set as follows: curtain gas (CUR): 15.0 psi; collision gas (CAD): medium; ion spray (IS) voltage: −4500 V; source temperature: 500 °C; GS1: 60 psi and GS2: 50 psi. The mass detector was programmed to perform two consecutive modes: enhanced mass scan (EMS) and multiple reaction monitoring—information-dependent acquisition—triggering enhanced product ion (MRM-IDA-EPI) mode analysis. EMS was employed to show full scan spectra to give an overview of all the ions in a sample. The settings used were: declustering potential (DP) −100 V, entrance potential (EP) −6 V, and collision energy (CE) −10 V. Spectra were recorded in negative ion mode between *m*/*z* 100 and 1000. MRM–IDA–EPI was performed, the IDA threshold was set at 200 counts per second, and the CE in EPI mode was set at −25 eV (CE spread of 10 eV). The EPI scan was operated at a scan rate of 4000 amu/s with dynamic fill in the linear ion trap. Various phenolic acids and flavonoids in EAW were identified by comparing the retention times and mass spectra with the reference standards.

### 2.8. Statistical Analysis

Experimental results are shown as mean ± SD. Statistical analysis was performed using Duncan’s multivariate test and the GLM (General Linear Model) of the SAS (Statistical Analysis System). Significant differences were defined as *p* < 0.05.

## 3. Results

### 3.1. EAW Prevents Body Weight Gain

**Figure 1:**
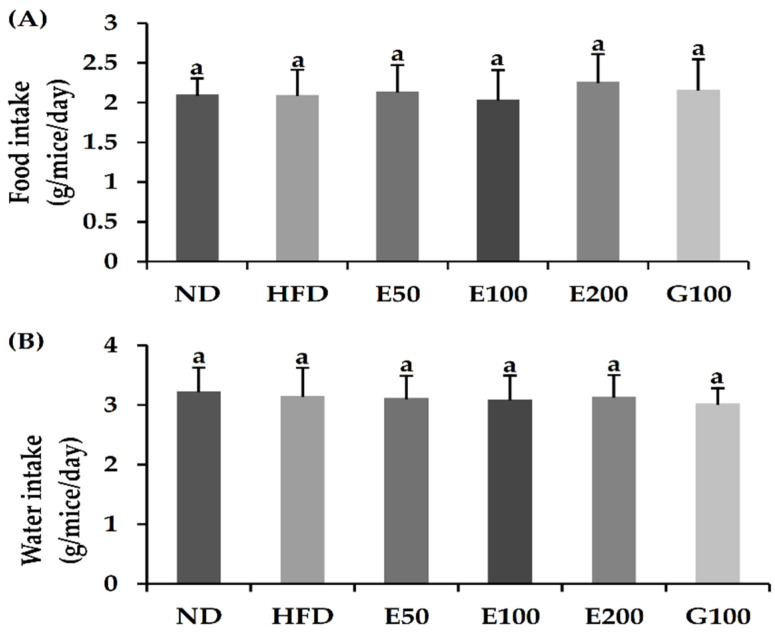
Effects of EAW on food intake (**A**) and water intake (**B**) in HFD-induced obese mice. Values are expressed as mean ± SD (*n* = 8). Different lowercase alphabets indicate that differences between means were considered statistically significant when *p* < 0.05. ND, normal diet mice group (10% fat); HFD, high-fat (60% fat) diet-induced obese mice group; E50/100/200, high-fat diet-induced obese mice were orally treated with *Erigeron annuus* (L.) Pers. water extract (EAW) at concentrations of 50/100/200 mg/kg/day; G100, high-fat diet-induced obese mice were orally treated with *Garcinia cambogia* water extract at concentrations of 100 mg/kg/day.

After 8 weeks, the bodyweights of HFD-fed mice significantly increased compared with ND-fed mice. The final body weight of the ND group was 33.03 ± 0.21 g, whereas the HFD group was 47.06 ± 0.25 g. After EAW treatment, the bodyweight of HFD-EAW-treated groups (50, 100, and 200 mg/kg/day) was considerably lower than that of the mice in the HFD group (Table 2). In particular, the bodyweight gain of the EAW 200 mg/kg/day treatment group was 32.28% lower than that of the HFD group. Compared with the ND and HFD groups, the dietary intake and water intake was not significantly different in any of the EAW-treated groups (Figure 1A,B).

### 3.2. Effect of EAW on Epididymal Adipose Tissue and Liver Mass Gain

**Figure 2:**
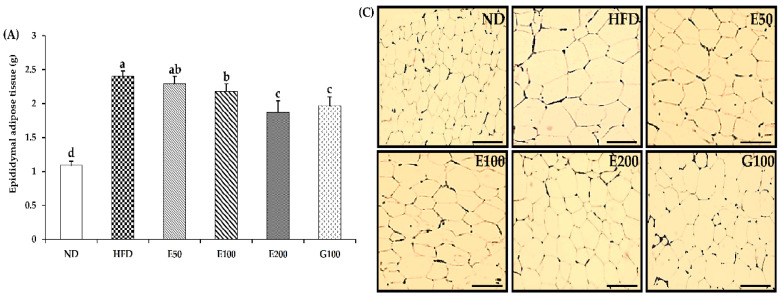

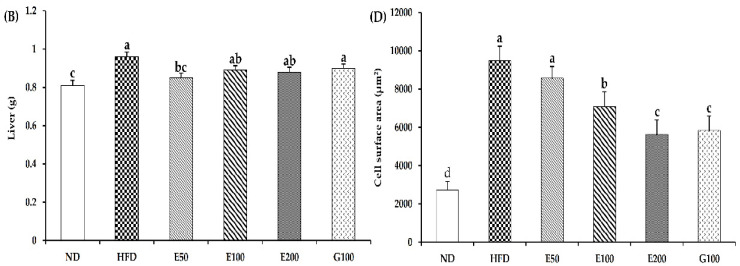
Effect of EAW on organ weight in HFD-induced obese mice. Epididymis adipose tissue (**A**) and liver (**B**) were obtained from the mice after fasting for 12 h at the end of the study. Epididymis adipose tissue was stained with hematoxylin and eosin and viewed under a microscope (400×) (**C**), and cell area was estimated by Adiposoft software (**D**). The scale bar depicts a length of 20 μm. Values are expressed as the mean ± SD (*n* = 8). Different lowercase alphabets indicate that differences between means were considered statistically significant when *p* < 0.05. ND, normal diet mice group (10% fat); HFD, high-fat (60% fat) diet-induced obese mice group; E50/100/200, high-fat diet-induced obese mice were orally treated with *Erigeron annuus* (L.) Pers. water extract (EAW) at concentrations of 50/100/200 mg/kg/day; G100, high-fat diet-induced obese mice were orally treated with *Garcinia cambogia* water extract at concentrations of 100 mg/kg/day.

Epididymis adipose tissue weight from HFD plus 100 and 200 mg/kg/day of EAW-treated mice was meaningfully reduced by 9.17% and 22.08%, respectively, compared with HFD-fed mice (Figure 2A). However, liver weights were not different between all the administration groups except for the HFD plus 50 mg/kg/day of EAW-treated group. (Figure 2B). Size of adipocytes in HFD-fed mice were distended compared with ND-fed mice, whereas HFD plus 100 and 200 mg/kg/day of EAW-treated mice significantly attenuated cell size. Among them, the area of adipocytes in the G100 and E200 groups decreased the most significantly by 38.38% and 40.52%, respectively (Figure 2C,D).

### 3.3. Effect of EAW on Serum Parameters

The serum TG and GLU levels of the HFD plus EAW 200 mg/kg/day group were significantly attenuated by 20.11% and 15.43% compared with HFD-fed mice (*p* < 0.05). Moreover, serum TC and LDL-c levels in mice fed HFD supplemented with 200 mg/kg/day of EAW were meaningfully reduced by about 14.20% and 37.93%, respectively, compared with HFD-fed mice (*p*  <  0.05). Nevertheless, as shown in Table 3, no significant changes in AST, ALT, and HDL-c levels were observed among the groups.

### 3.4. Effect of EAW on Adipogenic and Lipogenic Genes in Epididymal Adipose Tissue and Liver

**Figure 3:**
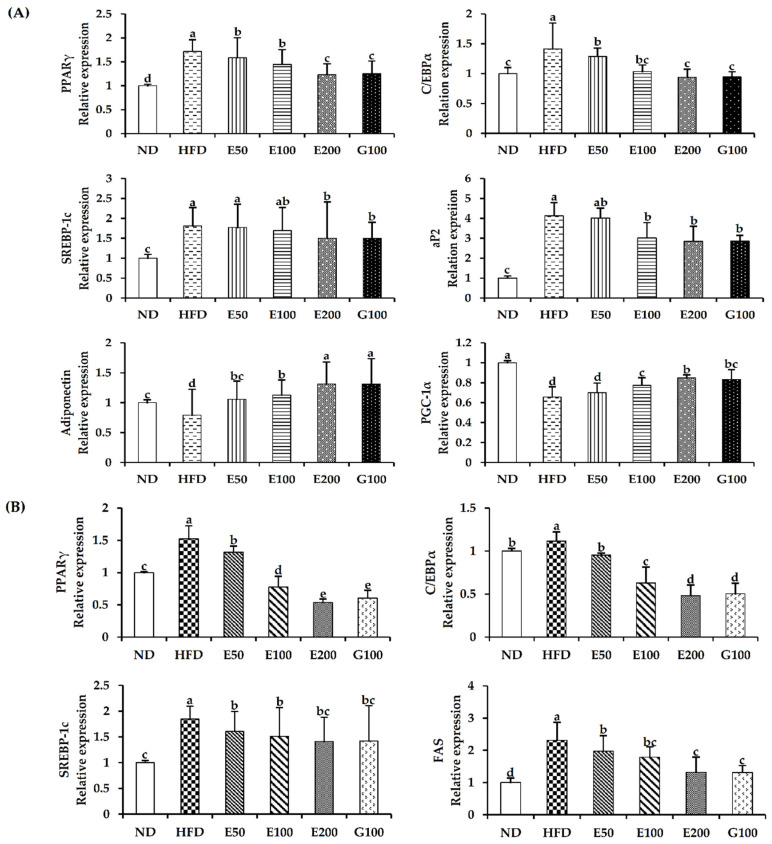
Effect of EAW on the expression of adipogenic and lipogenic genes in epididymis adipose tissues (**A**) and liver (**B**). Total RNA was extracted from epididymis adipose tissue and liver and subjected to real-time PCR analyses using primers specific for PPARγ, C/EBPα, aP2, FAS, PGC-1α, adiponectin, and SREBP-1c. Values are expressed as mean ± SD (*n* = 3). Different lowercase alphabets indicate that differences between means were considered statistically significant when *p* < 0.05. ND, normal diet mice group (10% fat); HFD, high-fat (60% fat) diet-induced obese mice group; E50/100/200, high-fat diet-induced obese mice were orally treated with *Erigeron annuus* (L.) Pers. water extract (EAW) at concentrations of 50/100/200 mg/kg/day; G100, high-fat diet-induced obese mice were orally treated with *Garcinia cambogia* water extract at concentrations of 100 mg/kg/day.

Expression of PPARγ and C/EBPα mRNA in the epididymal adipose tissue of HFD-fed mice was higher than in the EAW 200 mg/kg/day group. In the epididymis adipose tissue, the HFD plus EAW 200 mg/kg/day group showed that aP2 and SREBP-1c mRNA expression was significantly down-regulated compared with HFD-fed mice. The adiponectin and PGC-1α mRNA expressions of the HFD plus EAW 200 mg/kg/day group increased compared with HFD-fed mice (Figure 3A). Moreover, EAW significantly suppressed the mRNA expression of PPARγ, C/EBPα, and SREBP-1c in the liver at 200 mg/kg/day and significantly reduced the mRNA expression of the fat synthesis-related enzyme FAS (Figure 3B).

**Figure 4:**
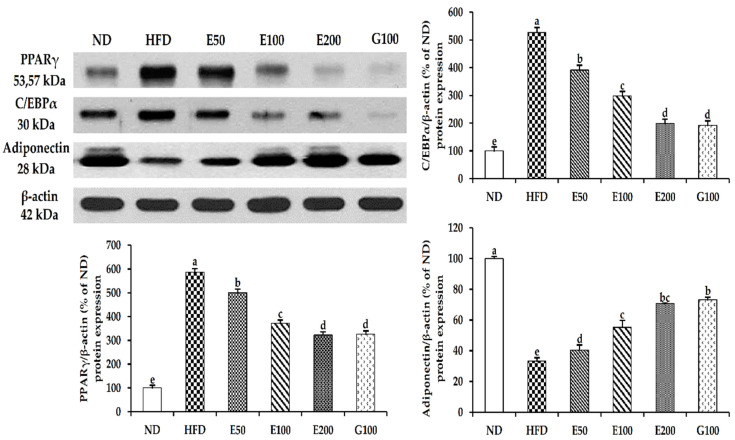
Effects of EAW on the levels of adipogenesis-related proteins in the liver. The liver was homogenized, and lysates were subjected to western blot analysis for PPARγ, C/EBPα, and adiponectin. The intensity of each band was quantified using ImageJ. Values and is expressed as mean ± SD (*n* = 3). Different lowercase alphabets indicate that differences between means were considered statistically significant when *p* < 0.05. ND, normal diet mice group (10% fat); HFD, high-fat (60% fat) diet-induced obese mice group; E50/100/200, high-fat diet-induced obese mice were orally treated with *Erigeron annuus* (L.) Pers. water extract (EAW) at concentrations of 50/100/200 mg/kg/day; G100, high-fat diet-induced obese mice were orally treated with *Garcinia cambogia* water extract at concentrations of 100 mg/kg/day.

We further investigated the protein levels of PPARγ, C/EBPα, and adiponectin in the liver. Figure 4 shows that the EAW treatments dose-dependently inhibited both PPARγ and C/EBPα protein levels, which correlated with the inhibition of the mRNA expression of both genes. Furthermore, the protein level of adiponectin in the liver treated with EAW significantly increased compared with the HFD group (Figure 4).

### 3.5. The AMPK Pathway of Activation by EAW in Epididymis Adipose Tissue

**Figure 5:**
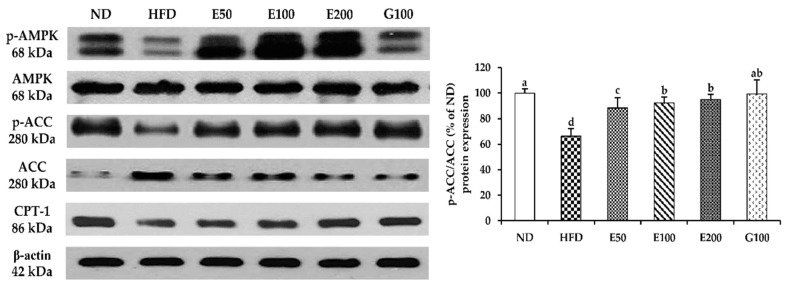

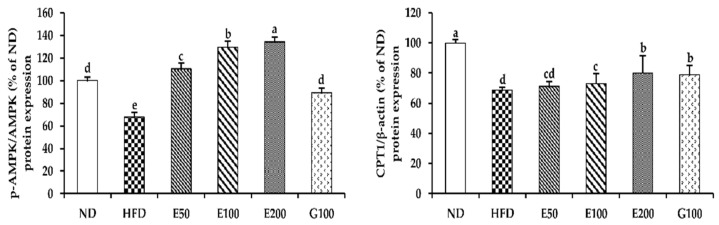
Effects of EAW on the activation of AMPK signaling in epididymis adipose tissues. Epididymis adipose tissue was homogenized, and the lysates were subjected to western blot analysis for p-AMPK, p-ACC, and CPT1. The intensity of each band was quantified using ImageJ. Values and is expressed as mean ± SD (*n* = 3). Different lowercase alphabets indicate that differences between means were considered statistically significant when *p* < 0.05. ND, normal diet mice group (10% fat); HFD, high-fat (60% fat) diet-induced obese mice group; E50/100/200, high-fat diet-induced obese mice were orally treated with *Erigeron annuus* (L.) Pers. water extract (EAW) at concentrations of 50/100/200 mg/kg/day; G100, high-fat diet-induced obese mice were orally treated with *Garcinia cambogia* water extract at concentrations of 100 mg/kg/day.

Western blot analysis demonstrated that EAW-administered groups enhanced AMPK and ACC phosphorylation in epididymal adipose tissue compared with HFD mice. EAW also significantly promoted the protein expression of CPT1 compared with HFD mice (Figure 5).

### 3.6. Identification of Phenolic Acids and Flavonoids in EAW

Since identification of phenolic acids and flavonoids in EAW was allowed by comparison of retention times, the observation of MRM transitions and MRM-IDA-EPI mode-mass fragmentation compare to commercial standards in LC-ESI-MS and LC-ESI-MS/MS. The negative ion mode of MRM-IDA-EPI mode produced the precursor ion [M − H]^−^, which was further analyzed by the product ion to produce a fragmentation pattern from the precursor ion.

**Figure 6:**
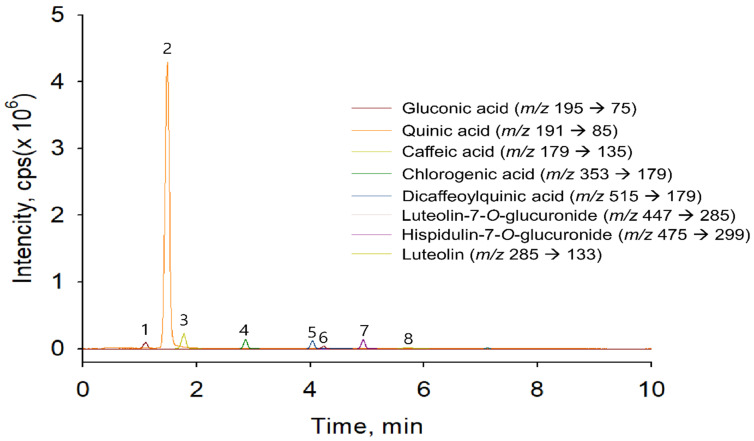
MRM chromatogram (TIC) of the *Erigeron annuus* (L.) Pers. water extract (EAW) by the LC-MS/MS method. Peak 1, gluconic acid; Peak 2, quinic acid; Peak 3, caffeic acid; Peak 4, chlorogenic acid; Peak 5, 3,4-dicaffeoylquinic acid; Peak 6, luteolin-7-*O*-glucuronide; Peak 7, hispidulin-7-*O*-glucuronide; Peak 8, luteolin.

Figure 6 shows the total ion chromatogram (TIC) of the extract EAW acquired in the negative ion mode using MRM detection. Table 4 summarizes the retention time, precursor ion, and mass fragmentation patterns of each component.

Peak 1, with a retention time of 1.11 min and *m*/*z* 195, was identified as gluconic acid. Detection of MRM-IDA-EPI mode for peak 1 was set with collision energy (CE) 25 V, and fragments *m*/*z* 177 [M − H-2H2O-CH2O]^−^ and *m*/*z* 129 [M − H-2H2O-CH2O]^−^ were observed. Peak 2 was found at a retention time of 1.52 min and *m*/*z* 191. In addition, fragment ions were found at *m*/*z* 173 [M-H2O-H]^−^, *m*/*z* 171 [M-H2O-H2-H]^−^, *m*/*z* 127 [M-H2O-CO2-H2-H]^−^, and *m*/*z* 109 [M-2H2O-CO2-H2-H]^−^. Peak 2 was identified as quinic acid compared with mass fragmentation patterns. Peak 3 was recorded at a retention time of 1.78 min and *m*/*z* 179. In EPI mode, mass fragment ions at *m*/*z* 135 [M-COO-H] ^−^ were identified as caffeic acid. Peak 4 with a retention time of 2.86 min was characterized as one of the known chlorogenic acid, caffeoylquinic acid, fragment ions at *m*/*z* 191[quinic acid], and *m*/*z* 173[caffeic acid] was corresponding. Peak 5 with a pseudomolecular ion at *m*/*z* 515 and product ions at *m*/*z* 353 [caffeoyl-quinic acid] and *m*/*z* 191 [quinic acid] were identified as 3,4-dicaffeoylquinic acid. Peak 6 was found at a retention time of 4.34 min and *m*/*z* 461. Product ions profiling with EPI scan mode of Peak 6 produced a precursor ion at *m*/*z* 461 and product ions at *m*/*z* 285 [M − H-glucuronide]^−^, *m*/*z* 175, 151, and 133 correspondings to luteolin aglycone and its mass fragmentation, revealing the compound to be luteolin-7-O-glucuronide. Peak 7 was recorded at a retention time of 4.95 min with *m*/*z* 475. Product ions profiling with the EPI scan mode of Peak 7 produced a precursor ion at *m*/*z* 475 and product ions at *m*/*z* 299 [M − H-glucuronide]^−^, *m*/*z* 284 [M − H-glucuronide-CH3]^−^, *m*/*z* 211 [M − H-glucuronide-CH3 -CHO-CO2]^−^, *m*/*z* 227 [M − H-glucuronide-CH3-CHO-CO]^−^, and *m*/*z* 255 [M − H-glucuronide-CH3-CHO]^−^. Peak 7 was identified as Hispidulin-7-O-glucuronide compared with mass fragmentation patterns, according to previous reports. Peak 8 was found at a retention time of 7.11 with *m*/*z* 285. Detection of MRM-IDA-EPI mode for Peak 8 was settled with CE 25V to fragment *m*/*z* 285 [M − H]^−^, *m*/*z* 241 [M-44 Da-H]^−^, *m*/*z* 217 [M-68 Da-H]^−^, *m*/*z* 199 [M-86 Da-H]^−^, *m*/*z* 175 [M-110 Da-H]^−^, *m*/*z* 151 [M-134 Da-H]^−^, and *m*/*z* 133 [M-152 Da-H]^−^. Peak 8 was characterized as luteolin compared with commercial reference standard and previous study fragmentation patterns. Our results are completely in agreement with previous studies and commercial standards.

## 4. Discussion

This study comprehensively investigated the effects of EA as an inexpensive functional food in preventing and treating obesity. Obese mice appeared healthy after dietary supplementation with EAW at various concentrations (50–200 mg/kg) without abnormal signs. There was no significant difference in serum ALT and AST levels among EAW-treated groups and the other groups (Table 3), indicating that oral EAW did not cause significant hepatotoxicity within 8 weeks.

EAW contains a variety of biologically active phenolic acids, including quinic acid, caffeic acid, chlorogenic acid, and 3,4-dicaffeoylquinic acid (Figure 6 and Table 4). Quinic acid, caffeic acid, and chlorogenic acid have been reported to inhibit adipogenesis and promote lipolysis in 3T3-L1 adipocytes [23]. Chlorogenic acid [24] and 3,4-dicaffeoylquinic acid [25] can activate the browning and thermogenesis of adipose tissue mediated by the AMPK/PGC1α pathway, while caffeic acid [26] can suppress the lipogenic transcription factor SREBP1c. Therefore, we speculate that the above-mentioned active substances may provide the anti-obesity effect of EAW.

SREBP-1c is a vital transcription factor related to the regulation of FAS and fatty acid biosynthesis-related genes to promote TG synthesis and regulate new fat [27]. EAW can reduce TG accumulation in the liver and adipose tissue by inhibiting the expression of SREBP-1c and FAS (Figure 3). PGC-1α (peroxisome proliferator-activated receptor coactivator-1α) regulates fat mass and fatty homeostasis. Generally, the expression level of PGC-1α in brown adipocytes is higher than that in white adipocytes [28]. EAW promotes the ectopic expression of PGC-1α (Figure 3), which could modulate metabolic disturbances, and promote the transition from white to brown adipocytes. This demonstrates that EAW can reduce the de novo synthesis of TG and cause browning of the white adipocytes that have formed to produce more heat to consume the deposited fat. PPARγ and C/EBPα play a vital role in the transcription of various genes involved in fat accumulation [29]. We found that the HFD plus EAW groups significantly (*p*  <  0.05) attenuated the expression of PPARγ and C/EBPα in epididymal fat tissue and liver (Figure 3 and Figure 4). These transcription factors regulate adipogenic genes (such as ap2) to regulate lipid synthesis and accumulation [30]. EAW attenuates the central hypertrophy of adipocytes by inhibiting the synthesis and accumulation of lipids and reduces the mass of epididymal adipose tissue, which ultimately promotes bodyweight loss (Table 2 and Figure 2).

Adiponectin is one of the critical adipocytokines specifically expressed in white adipose tissue. It is considered one type of AMPK agonist that combines energy homeostasis with insulin action [31]. Increased adiponectin hormone levels are associated with weight loss in obese animals [32]. HFD plus EAW groups were shown to increase adiponectin levels in epididymis adipose tissues compared with the HFD mice (Figure 3). A significant increase in adiponectin expression seems to indicate that EAW can activate the AMPK pathway. AMPK is a catabolism signaling substance that can burn fat and promote glucose absorption by muscle cells. This pathway is related to modulation of lipid metabolism, body weight, mitochondrial biosynthesis, and insulin signaling [33]. Activation of AMPK rapidly reverses the synthesis of fatty acids through phosphorylation and inactivation of ACC and reduces malonyl-CoA production (as an allosteric inhibitor of CPT1) [34]. CPT1 regulates acyl-CoA entry and β-oxidation in the mitochondrial outer membrane [35]. AMPK regulates lipid metabolism by regulating triglyceride synthesis and fatty acid oxidation, which appears to be inseparable from SREBP-1c downstream of AMPK [36]. However, AMPK phosphorylation in C57BL/6J mice was inhibited by HFD [37]. The absence of AMPK or its reduced activity results in diminished biogenesis, fatty acid oxidation, and hepatic glucose uptake in mitochondria [7]. In contrast, activation of AMPK by various means was shown to restore normal blood glucose [38] and reduce hepatic glucose production [39] and plasma TG levels in the obese animal model [40]. EAW decreased the activity of ACC by phosphorylating AMPK while significantly increasing the protein level of CPT1 (Figure 5). The results of this study suggest that EAW may reduce lipid accumulation in liver and epididymal adipose tissue by promoting fatty acid oxidation mediated by AMPK activation regulation. The increased mRNA level of PGC-1α also shows that EAW can increase the energy consumption process by producing heat (Figure 3). Therefore, we observed that EAW significantly reduced serum hyperlipemia and hyperglycemia caused by HFD (Table 3).

## 5. Conclusions

Although further studies are needed to determine whether the compounds in EAW have a synergistic effect and the mechanism by which it activates the AMPK pathway, based on these present results, EAW is a potential dietary intervention tool that can be used to prevent weight gain and treat metabolic syndromes. The overall conclusion is that EA may be of potential value for use as a natural anti-obesity substance and therefore a functional food.

## Figures and Tables

**Table 1:** Primer sequences used for real-time PCR.

Gene Name	Accession No.	Forward Primer	Reverse Primer
PPARγ	NM_011146	5’-CGCTGATGCACTGCCTATGA-3’	5’-AGAGGTCCACAGAGCTGATTCC-3’
C/EBPα	BC058161	5’-CGCAAGAGCCGAGATAAAGC-3’	5’-CACGGCTCAGCTGTTCCA-3’
aP2	NM_024406	5’-CATGGCCAAGCCCAACAT-3’	5’-CGCCCAGTTTGAAGGAAATC-3’
FAS	NM_007988	5’-CTGAGATCCCAGCACTTCTTGA-3’	5’-GCCTCCGAAGCCAAATGAG-3’
PGC-1α	NM_008904	5’-TGTTCCCGATCACCATATTCC-3’	5’-GGTGTCTGTAGTGGCTTGATTC-3’
Adiponectin	NM_009605	5’-ATCCACACGTGTACTCAC-3’	5’-AGCATGGTCTACTTCCAG-3’
SREBP-1c	NM_011480.3	5’-GGACGAGCTGGCCTTCGGTGA-3’	5’-ATAGGGGGCGTCAAACAGGCCC-3’
GAPDH	BC083080	5’-GTATGACTCCACTCACGGCAAA-3’	5’-GGTCTCGCTCCTGGAAGATG-3’

C/EBPα, CCAAT/enhancer-binding proteinα; PPARγ, peroxisome proliferator-activated receptorγ; SREBP-1c, sterol regulatory element-binding protein-1c; FAS, fatty acid synthase; aP2, adipocyte fatty acid-binding protein 2.

**Table 2:** Effect of EAW in HFD-induced obese mice on body weight.

Parameters	Groups					
	ND	HFD	E50	E100	E200	G100
Initial body weight (g)	24.06 ± 0.19 ^a^	24.16 ± 0.38 ^a^	24.08 ± 0.21 ^a^	24.09 ± 0.39 ^a^	24.09 ± 0.23 ^a^	24.11 ± 0.21 ^a^
Final body weight (g)	33.03 ± 1.21 ^d^	47.06 ± 2.25 ^a^	43.26 ± 1.51 ^b^	42.04 ± 1.78 ^b^	39.05 ± 1.51 ^c^	40.08 ± 1.89 ^c^
Body weight gain (g)	8.97 ± 1.47 ^d^	22.09 ± 2.29 ^a^	19.18 ± 0.63 ^b^	17.95 ± 1.54 ^b^	14.96 ± 1.49 ^c^	15.97 ± 1.74 ^c^

Values are expressed as the mean ± SD (*n* = 8). Different lowercase alphabets indicate that differences between means were considered statistically significant when *p* < 0.05. ND, normal diet mice group (10% fat); HFD, high-fat (60% fat) diet-induced obese mice group; E50/100/200, high-fat diet-induced obese mice were orally treated with *Erigeron annuus* (L.) Pers. water extract (EAW) at concentrations of 50/100/200 mg/kg/day; G100, high-fat diet-induced obese mice were orally treated with *Garcinia cambogia* water extract at concentrations of 100 mg/kg/day.

**Table 3:** Effects of EAW on biochemical serum parameters in HFD-induced obese mice.

Serum	Group					
	ND	HFD	E50	E100	E200	G100
TC (mg/dL)	109.73 ± 4.01 ^c^	169.69 ± 2.212 ^a^	152.31 ± 9.89 ^ab^	152.23 ± 10.03 ^ab^	145.60 ± 5.165 ^b^	142.28 ± 1.235 ^b^
TG (mg/dL)	57.63 ± 3.77 ^c^	94.71 ± 4.80 ^a^	91.82 ± 3.41 ^a^	87.98 ± 3.22 ^ab^	75.66 ± 6.59 ^b^	76.42 ± 8.90 ^b^
HDL-C (mg/dL)	78.249 ± 1.24 ^b^	116.13 ± 3.21 ^a^	119.88 ± 3.32 ^a^	121.04 ± 4.12 ^a^	138.82 ± 2.99 ^a^	139.07 ± 3.11 ^a^
LDL-C (mg/dL)	10.06 ± 1.72 ^c^	31.24 ± 2.11 ^a^	31.14 ± 2.76 ^a^	29.87 ± 1.86 ^a^	19.39 ± 1.96 ^b^	20.31 ± 1.24 ^b^
AST (U/L)	127.43 ± 21.23 ^a^	119.04 ± 11.25 ^a^	119.64 ± 27.06 ^a^	127.22 ± 18.33 ^a^	95.19 ± 6.34 ^a^	95.16 ± 4.33 ^a^
ALT (U/L)	31.96 ± 3.45 ^a^	43.24 ± 2.11 ^a^	41.76 ± 3.25 ^a^	39.21 ± 2.31 ^a^	28.93 ± 1.66 ^a^	28.31 ± 1.32 ^a^
GLU (mg/dL)	146.38 ± 4.12 ^c^	178.13 ± 3.99 ^a^	165.09 ± 10.01 ^ab^	170.88 ± 9.74 ^ab^	150.65 ± 4.55 ^b^	155.34 ± 5.23 ^b^

Values are expressed as mean ± SD (*n* = 8). Different lowercase alphabets indicate that differences between means were considered statistically significant when *p* < 0.05. Aspartate aminotransferase (AST); alanine aminotransferase (ALT); total cholesterol (TC); glucose (GLU); high-density lipoprotein (HDL-c); low-density lipoprotein (LDL-c); triglyceride (TG). ND, normal diet mice group (10% fat); HFD, high-fat (60% fat) diet-induced obese mice group; E50/100/200, high-fat diet-induced obese mice were orally treated with *Erigeron annuus* (L.) Pers. water extract (EAW) at concentrations of 50/100/200 mg/kg/day; G100, high-fat diet-induced obese mice were orally treated with *Garcinia cambogia* water extract at concentrations of 100 mg/kg/day.

**Table 4:** Summarization of mass spectra data of flavonoids and polyphenols in EAW.

Peak	Retention Time (min)	[M − H]^−^	Mass Fragmentation	Compound Name	References
1	1.11	195	177, 147, 129, 111, 99, 75	Gluconic acid	Li et al. [19] Felipe et al. [20]
2	1.52	191	173, 171, 127, 109, 93, 85, 65	Quinic acid	Fathoni et al. [21]
3	1.78	179	135, 107, 89, 59	Caffeic acid	Li et al. [19]
4	2.86	353	191, 179, 161, 135, 93, 85	Chlorogenic acid	Li et al. [19]
5	4.04	515	353, 85, 93, 135, 173, 179, 191, 161	3,4-Dicaffeoylquinic acid	Simirgiotis et al. [22]
6	4.34	461	285, 133, 151, 175	Luteolin-7-*O*-glucuronide	Li et al. [19]
7	4.95	475	299, 284, 211, 227, 255	Hispidulin-7-*O*-glucuronide	Li et al. [19]
8	7.11	285	241, 217, 199, 175, 151, 133	Luteolin	Fathoni et al. [21]

Various phenolic acids and flavonoids in *Erigeron annuus* (L.) Pers. water extract (EAW) were determined by comparing retention time and mass spectra with reference standards. Peak number, refer to Figure 6.
